# Promotion or Prevention Messaging?: A Field Study on What Works When You Still Have to Work

**DOI:** 10.3389/fpsyg.2018.01990

**Published:** 2018-10-17

**Authors:** Marta Anna Roczniewska, E. Tory Higgins

**Affiliations:** ^1^Sopot Faculty of Psychology, SWPS University of Social Sciences and Humanities, Sopot, Poland; ^2^Department of Psychology, Columbia University, New York City, NY, United States

**Keywords:** information framing, reference points, regulatory focus theory, regulatory fit, organizational change, Sunday trade ban

## Abstract

This article addresses the timely subject of the reactions toward a Sunday trade ban in Poland. The law introduced in March 2018 created a division among service employees: (1) those who used to work on Sundays before the law and now enjoy work-free Sundays, and (2) those who used to work and still have to work on Sundays. Although the objective circumstances did not change for this latter group, their current status quo (0) now had a new better state (+1) as a contrast reference point. Hence, this group experiences a non-gain rather than a loss. Using the framework of regulatory focus (Higgins, 1997) and regulatory fit (Higgins, 2000) theories, we predicted that the individuals in the non-gain condition would process a promotion (vs. prevention)-framed message more fluently. We also predicted that processing fluency would enhance fairness perceptions among these employees. To test these predictions, we conducted a field experiment, manipulating message framing among two groups of service employees: those who gained and those who did not gain as a result the Sunday trade ban. Analysis of variance revealed that employees in a non-gain promotion framing condition processed the message more fluently than those in a prevention framing condition. A moderated mediation analysis also showed that the processing fluency resulting from fit created higher fairness perceptions. Repercussions for communicating about organizational non-gain changes are discussed.

## Introduction

“This law isn’t really just^1^"—a 29-year-old tram driver from Warsaw complained about the newly introduced bill that banned trade on Sundays in Poland (**Supplementary Material 1**). His upset resulted from the fact that the ban ‘relieved’ shop employees who experienced a gain from this change while some other professionals (like himself) still had to work on Sundays. Interestingly, his *objective* work situation did not change–he works on several Sundays just as he used to. However, this law created a better state (+1) as a new contrast reference point (no work on Sundays) that made his current status quo (0) a less valued option. This new reference point made his current condition, psychologically, a negative non-gain (Higgins and Liberman, 2018).

The purpose of our research was to examine the role of change communication in determining fairness perceptions among individuals who experience change as a non-gain. To the best of our knowledge, no previous research has investigated the consequences of a policy transformation for change that benefited some while maintaining the status quo for others. This seems important because the perspective of the group whose status quo is maintained may be neglected in messaging about the change because their objective circumstances have not changed. Their psychological perceptions, however, including their experience of unfairness, could be negatively affected (Roczniewska et al., 2018). Thus, it is important to consider what could be done about this.

### The Status Quo “0”

The existing state of affair, i.e., the status quo “0,” is a reference point for determining the value of the events (Kahneman and Tversky, 1979). Importantly for the current research issue, individuals may represent and appraise the same status quo “0” differently, depending on other reference points (Higgins, 2018; Higgins and Liberman, 2018). Persons who contrast it with a better “+1” state—a gain experience their current situation negatively because it represents an absence of a positive outcome–a non-gain. We propose that introducing the Sunday trade ban created a condition of a non-gain among those professionals who, after the law was introduced, still had to work on Sundays because of legislator exclusions, including restaurant, gas-station, or public transportation employees. Their status quo “0” was contrasted with a new, better “+1” state of not working on Sunday, i.e., a non-gain for them, which could create perceptions of unfairness, like that expressed by the tram driver. A non-gain places people in a promotion focus. A message about the change that suits this promotion psychological condition would be more engaging, which in turn could make the message more effective (Avnet et al., 2013). Let’s now consider how this could work.

### Communicating Non-gains

Can perceptions of unfairness be alleviated? We propose that change messages that address the non-gain situation properly with arguments corresponding to this promotion-related situation can improve fairness perceptions. Regulatory Focus Theory (Higgins, 1997) distinguishes between two systems of goal pursuit: promotion and prevention. The promotion system is concerned with attaining gains, whereas the prevention system is concerned with maintaining non-losses (Higgins, 1997). Promotion is concerned with growth and advancement, whereas prevention is concerned with safety and security (Higgins, 1997). Promotion-focused individuals represent goals as ideals and aspirations, whereas individuals high in prevention represent goals as oughts and responsibilities (Higgins, 1997).

Given this, a non-gain state would be better addressed with promotion than prevention message framing, a promotion message concerned with ideals, advancement, and possible gains (Cesario et al., 2008). Additionally, we argue that a non-gain condition activates self-regulation processes to move toward a “+1” state, while for individuals in a gain condition this need is already satisfied, and thus this type of persuasive communication is less motivating, which has been found when promotion-focused individuals have already experienced advancement (Zou et al., 2014). This is also consistent with the view that the relevance of the objects changes along with the motivational priorities of the individual (Lewin, 1935; Ferguson and Bargh, 2004).

### From *Fit* to *Fair*

Evidence suggests that when a framing of a message matches the motivational orientation of its recipient, it is processed more fluently (Lee and Aaker, 2004). Studies demonstrate that persuasive communication is attended more easily in a fit than a non-fit condition (Cesario and Higgins, 2008); it also seems more comprehensible (Zhao and Pechmann, 2007) and engaging (Pierro et al., 2013) to its recipients. Hence, we predict that:

*Hypothesis 1.* Promotion message framing is processed more fluently than prevention message framing when one’s reference point is a non-gain condition.

Previous research has demonstrated that processing fluency explains the positive effect of regulatory fit on persuasion (Lee and Aaker, 2004). The ease and comprehensibility influence judgments because feelings act as decision heuristics (Schwarz, 2002), especially when individuals are not motivated to process the information thoroughly (Avnet et al., 2013). Ample research demonstrates that regulatory fit “feels right” (Cesario et al., 2004), and this experience can transfer to judgments: “what *feels* right, *is* right” (Camacho et al., 2003; Higgins, 2005). Therefore, processing fluency may contribute to the “feeling right” phenomenon when individuals are exposed to persuasive messages that match their orientation. Interestingly, research has demonstrated that regulatory fit increases fairness perceptions (Li et al., 2011; Roczniewska et al., 2018). When individuals read decision justifications that match their focus, they deem the situation more just (Roczniewska and Higgins, unpublished). We propose that message processing fluency, derived from regulatory fit, enhances fairness perceptions:

*Hypothesis 2.* Regulatory fit predicts higher fairness perceptions via better processing fluency.

## Method

### Participants and Procedure

The study was approved by the local Ethics Board and it was conducted in April 2018 (2nd month of the ban). The data was gathered using a pen-and-paper booklet containing experimental materials and scales. Four Research Assistants (RAs) approached employees working in distinct service points (stores, restaurants, gas stations, etc.) in Tricity area to seek their agreement to participate in the study about a Sunday trade ban. Participation was voluntary and anonymous. Overall, 201 employees filled in the booklets provided, of which 50 participants were in the “gain” condition (had to work before and don’t have to work now), 78 were in the “non-gain” group (had to work before and have to work now), and 73 were “neither” (did not work on Sundays before and after the bill). Because the research question concerned the comparison between the gain and non-gain conditions, we excluded the “neither” participants, leaving the total sample of 128 participants. Most of the participants were women (*n* = 86; 67% of the sample). Participants’ age ranged from 18 to 53 (*M* = 24.40; *SD* = 6.38).

#### Materials

We gathered demographic data and asked employees to state: (1) whether they used to work on Sundays before the trade ban was effective; and (2) whether they have to work on Sundays now that the bill is effective. These two questions allowed us to differentiate between a gain (coding = 1) and a non-gain (coding = 0) condition. The procedure started with a situational regulatory focus manipulation using a message describing the Sunday trade ban. Next, participants filled in two scales.

#### Message Framing

We randomly assigned participants to one of the two framing conditions. In *promotion* framing, the Sunday trade ban was described as being in line with family *ideals*. We argued that it allows *developing* family bonds and *promoting more* family time. In *prevention* framing, the ban was described as being in line with family *responsibilities*. We argued that it allows *protecting* family bonds and *securing* family time.

#### Message Fluency (α = 0.90)

We used 3 items developed by Lee and Aaker (2004) to measure the perceived fluency of the communication. Using an answering scale from 1 to 7, participants assessed the ease of processing, comprehensibility, and clarity of the message.

#### Perceptions of Change Fairness (α = 0.92)

We used a 7-item procedural justice subscale of the organizational justice measure (Colquitt, 2001). The items were adjusted to describe the ban (e.g., “The decision to introduce Sunday trade ban was free of bias”). Participants rated their agreement statements using a scale ranging from 1 (definitely not) to 5 (definitely yes).

## Results

**FIGURE 1:**
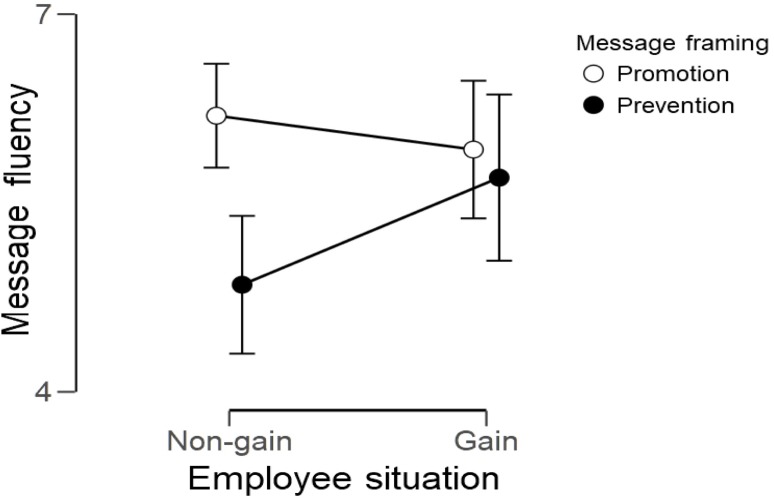
Interaction of the message framing and employee situation for message fluency. Error bars represent standard errors.

Hypothesis 1 predicted a matching effect; specifically, promotion framing is processed more fluently than prevention framing when one’s reference point is a non-gain. To test this prediction, we conducted a 2 (message framing: promotion vs. prevention) × 2 (employee situation: gain vs. non-gain) analysis of variance for processing fluency. **Figure 1** presents the observed interaction, *F*(1,124) = 3.77, *p* = 0.055, η^2^ = 0.03.

In line with the expectations, for employees in a non-gain condition, promotion framing was processed more fluently (*M* = 6.19, *SD* = 1.12) than prevention framing (*M* = 4.85, *SD* = 1.86), *p* < 0.001. For those in a gain condition, promotion (*M* = 5.92, *SD* = 1.49) and prevention (*M* = 5.70, *SD* = 1.37) framing were processed similarly, *p* = 0.622. Prevention message framing related to worse processing fluency in non-gain versus gain (*p* = 0.045) condition, while we observed no such difference for promotion framing (*p* = 0.496).

To test Hypothesis 2, we conducted a moderated mediation analysis (Model 7, Hayes, 2013) using SPSS 24 with the PROCESS macro, applying Bootstrapping 10,000 with bias-corrected confidence intervals for estimating indirect effects and a moderated mediation index (Preacher and Hayes, 2004). According to Hypothesis 2, regulatory fit predicts higher fairness perceptions via better processing fluency. The CI of the index of moderated mediation excluded 0, evidencing moderated mediation; estimate = −0.1157, SE = 0.0916, 95% CI [−0.3751, −0.0005]. In line with our predictions, the positive indirect effect of promotion framing on fairness perceptions via processing fluency was significant for a non-gain condition [estimate = 0.1387, SE = 0.0882, 95% CI (0.0024, 0.3570)], whereas it was not significant for a gain condition [estimate = 0.0230, SE = 0.0483, 95% CI (−0.0498, 0.1615)]. This supports Hypothesis 2.

## Discussion

Capitalizing on a recent event, this research engaged in theory testing in a field experiment regarding the important issue of how to message organizational change. When studying negative outcomes of an organizational change, most previous research has concerned losses, such as downsizing, benefit loss, salary cuts. In this research, we extended issues regarding organizational change by addressing a different but significant issue: what happens when the status quo is objectively the same after the change but is experienced as a negative condition because it is contrasted with a better “+1” reference point—a non-gain? Our study demonstrated that it matters how the change is framed in the messaging. Namely, a non-gain activates promotion orientation, and as such it is better addressed with messages focusing on ideals and advancement, than responsibilities and safety. A message that matches an individual’s orientation is processed more fluently, and this processing ease leads to higher fairness perceptions. We add another heuristic to fairness judgments (Cropanzano et al., 2001)–processing ease resulting from regulatory fit.

The fact that this is a single-study paper limits the strength of our conclusions. Many potential participants declined to take part in this research because the subject of the study was controversial. This resulted in a relatively small sample size, which put at risk the power of the study to detect the interaction that was small in size. Also, all participants worked in a relatively big municipal area, and the repercussions of the Sunday trade ban could be experienced differently in smaller than larger cities. This limits the generalizability of the outcomes we observed. A conceptual replication with a more representative and larger sample would be beneficial to better understand the phenomenon we explored. Finally, we expected that all participants who did not have to work on Sundays anymore would be satisfied with this outcome. However, it is possible that for some of them (e.g., University students) weekends are the only opportunity to work. The potential homogeneity of self-interests in the ‘gain’ group should also be explored in future studies.

This research investigated how to improve perceptions of unfairness from an organization change that is experienced as a non-gain because the current status quo “0” is contrasted with a new and better reference point “+1.” Future research could investigate change recipients in a loss versus non-loss state. Here, we expect that prevention rather than promotion message framing could mitigate the low fairness perceptions in the loss condition. Moreover, here the Sunday trade ban divided employees into those who benefited and those who did not, creating the gain versus non-gain perspective. However, research demonstrates that individuals have chronic dispositions to pursue gains or ensure against non-losses, and hence view the status quo differently (Higgins, 1997). Future studies should investigate the moderating role of individual self-regulatory orientations on the findings reported here.

## Ethics Statement

This study was carried out in accordance with the recommendations of Departamental Ethics Committe, SWPS University of Social Sciences and Humanities, Faculty in Sopot, with written informed consent from all subjects. All subjects gave written informed consent in accordance with the Declaration of Helsinki.

## Author Contributions

MR and EH developed the theory and wrote the paper. MR designed the study, collected the data, and performed data analysis and interpretation.

## Conflict of Interest Statement

The authors declare that the research was conducted in the absence of any commercial or financial relationships that could be construed as a potential conflict of interest.

## Footnotes

1 https://www.voanews.com/a/most-stores-shut-in-poland-as-sunday-trade-ban-takes-effect/4291603.html
