# Supplementary material for: Promotion or Prevention Messaging?: A Field Study on What Works When You Still Have to Work
Source: Front Psychol. 2018 Oct 17;9:1990. doi: 10.3389/fpsyg.2018.01990 (PMC6199381; doi:10.3389/fpsyg.2018.01990)
Supplement: Supplementary file 1 [file Table_1.DOCX]

**Supplementary material 1**

**Sunday trade ban–change context**

In March 2018 Poland imposed a ban on Sunday trade, with shopping malls, supermarkets and most other retailers closed for the first time since 1990s. This change is gradual, as—at first—trade is banned on two Sundays a month, rising to three Sundays from 2019, and finally all Sundays from 2020. This bill was propositioned by the trade union Solidarity, which argued that employees deserve Sundays off. However, this bill applies only to shop employees, as others—like restaurant, public transport, or emergency healthcare employees—still work on Sundays. The current research addresses this division of service workers between those, who used to work on Sundays before the law was introduced and now enjoy work-free Sundays (gain) and those who used to work and still have to work on Sundays (non-gain).
